# Distribution Patterns of Odonate Assemblages in Relation to Environmental Variables in Streams of South Korea

**DOI:** 10.3390/insects9040152

**Published:** 2018-10-29

**Authors:** Da-Yeong Lee, Dae-Seong Lee, Mi-Jung Bae, Soon-Jin Hwang, Seong-Yu Noh, Jeong-Suk Moon, Young-Seuk Park

**Affiliations:** 1 Department of Biology, Kyung Hee University, Seoul 02447, Korea; hg99228@hanmail.net (D.-Y.L.); dleotjd520@naver.com (D.-S.L.); 2 Freshwater Biodiversity Research Bureau, Nakdonggang National Institute of Biological Resources, Sangju, Gyeongsangbuk-do 37242, Korea; mjbae@nnibr.re.kr; 3 Department of Environmental Health Science, Konkuk University, Seoul 05029, Korea; sjhwang@konkuk.ac.kr; 4 Water Environment Research Department, Watershed Ecology Research Team, National Institute of Environmental Research, Incheon 22689, Korea; nsy2809@korea.kr (S.-Y.N.); waterfa@korea.kr (J.-S.M.); 5 Department of Life and Nanopharmaceutical Sciences, Kyung Hee University, Seoul 02447, Korea

**Keywords:** multivariate analysis, stream community, community analysis, indicator species, self-organizing map (SOM), non-metric multidimensional analysis (NMDS), freshwater ecology, Odonata

## Abstract

Odonata species are sensitive to environmental changes, particularly those caused by humans, and provide valuable ecosystem services as intermediate predators in food webs. We aimed: (i) to investigate the distribution patterns of Odonata in streams on a nationwide scale across South Korea; (ii) to evaluate the relationships between the distribution patterns of odonates and their environmental conditions; and (iii) to identify indicator species and the most significant environmental factors affecting their distributions. Samples were collected from 965 sampling sites in streams across South Korea. We also measured 34 environmental variables grouped into six categories: geography, meteorology, land use, substrate composition, hydrology, and physicochemistry. A total of 83 taxa belonging to 10 families of Odonata were recorded in the dataset. Among them, eight species displayed high abundances and incidences. Self-organizing map (SOM) classified sampling sites into seven clusters (A–G) which could be divided into two distinct groups (A–C and D–G) according to the similarities of their odonate assemblages. Clusters A–C were characterized by members of the suborder Anisoptera, whereas clusters D–G were characterized by the suborder Zygoptera. Non-metric multidimensional scaling (NMDS) identified forest (%), altitude, and cobble (%) in substrata as the most influential environmental factors determining odonate assemblage compositions. Our results emphasize the importance of habitat heterogeneity by demonstrating its effect on odonate assemblages.

## 1. Introduction

The distribution and abundance of organisms are governed by various environmental conditions [1], and thus, biodiversity is closely related to habitat heterogeneity as determined by these highly variable environmental factors [2]. The order Odonata includes both dragonflies and damselflies and is widely distributed across much of the world, being found in habitats ranging from the alpine mountains to tropical rainforests. Similarly, larval odonates are known to inhabit diverse environments including temporary and permanent water bodies in freshwater ecosystems [3]. Despite the wide habitat range of the members of Odonata, individual species can be clearly distinguished by their habitat preferences with most species being incredibly sensitive to environmental changes, particularly those caused by human activity [4]. Because of this sensitivity, odonates are commonly used as indicators of habitat disturbance [5,6,7]. In addition, they act as intermediate predators, connecting invertebrates and vertebrates in local ecosystem food webs [8], and providing a valuable ecosystem service by feeding on many insect pests [9]. Foote and Hornung [10] suggested that the diversity and abundance of larval odonates in ecosystems are highly representative of the diversity and abundance of local macroinvertebrates as a whole.

The distribution patterns of odonate assemblages are affected by various habitat characteristics. Changes in odonate biodiversity are directly related to urbanization and pollutant concentrations in water [11], and both landscape patterns and water body types can have strong effects on the structure of the Odonata communities [12]. Hofmann and Mason [13] found that larval odonates are directly affected by microhabitat conditions such as water velocity and biochemical oxygen demand, whereas adults are typically affected by larger-scale habitat characteristics, such as variation in land use and riparian vegetation. Similarly, Osborn and Samways [5] demonstrated that sunlight (primary production) and vegetation gradients were important in determining the patterns of adult dragonfly assemblages.

Although the distribution patterns of Odonata have been well studied in relation to their associated habitat conditions, most studies have been conducted only at local or regional scales [14,15], and few studies have investigated Odonata distribution patterns at larger scales, particularly in Asia, and more specifically in Korea. In South Korea, there are three endemic species (*Anisogomphus coreanus*, *Asiagomphus melanopsoides*, and *Nihonogomphus minor*) and three endangered species (*Nannophya pygmaea*, *Macromia daimoji*, and *Libellula angelina*) among the 102 recorded species of Odonata [16]. Odonata was mentioned as part of benthic macroinvertebrates in large-scale studies [17,18]. However, few studies have focused on investigating the distribution patterns of Odonata and the relationship between their occurrence patterns and variations in environmental conditions in aquatic ecosystems. Therefore, in this study, we aimed: (i) to investigate the distribution patterns of Odonata in streams across South Korea; (ii) to evaluate the relationship between the distribution patterns of Odonata and environmental conditions; and (iii) to identify indicator species and the most significant environmental factors that affect the distributions of these species.

## 2. Materials and Methods

### 2.1. Ecological Data

Data on odonate assemblages was obtained from the database of the National Aquatic Ecological Monitoring Program (NAEMP) maintained by the Ministry of Environment and the National Institute of Environmental Research, Korea. Benthic macroinvertebrates have been sampled at 1158 sites in South Korea during spring and autumn of every year since 2008, including the streams and rivers of the five major river catchments (Han, Nakdong, Geum, Seomjin, and Yeongsan) and the Jeju stream system. A Surber net (30 cm × 30 cm, 1 mm mesh size) was used for three replicate samplings of aquatic insects at each sampling site according to NAEMP protocol [19]. Samples were fixed with 95% alcohol and most specimens were identified to the species level [20]. The three replicate samples were pooled and used to calculate the number of individuals per square meter.

**Figure 1:**
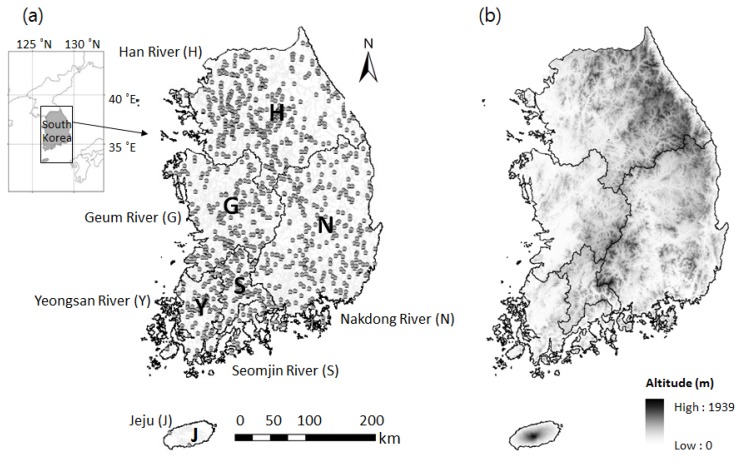
(**a**) Location of sampling sites; and (**b**) altitudinal map of South Korea.

From the database, we extracted data on odonate assemblages consisting of samples from 965 sites taken from 2009 to 2016, and used the average abundance values for each taxon in our analysis. The 965 sampling sites were evenly distributed throughout Korea (358, 259, 167, 107, and 72 sites in the Han, Nakdong, Geum, and Seomjin river catchments and the 2 Jeju Island sites, respectively) (Figure 1). The Han River (stream length: 8581 km, catchment area: 31,648 km²), located in the northern part of South Korea, has the largest catchment area among the five major catchments [21], and the Nakdong River (stream length: 9621 km and catchment area: 29,987 km²), located in the south eastern part of South Korea, has the second largest catchment area. The Geum River (stream length: 6126 km and catchment area: 15,959 km²) is located in the mid-west of South Korea at a relatively low altitude. The Yeongsan (stream length: 1274 km and catchment area: 5912 km²) and Seomjin (stream length: 2633 km and catchment area: 6588 km².) Rivers are located in southwestern Korea. Jeju Island (stream length: 605 km and catchment area: 966 km^2^) is home to several small temporary stream systems [22].

We measured 34 environmental variables at each site, which were grouped into six categories: geography, meteorology, land use, substrate composition, hydrology, and physicochemistry (Table 1). Variables classified into the geography, meteorology, and land use categories were extracted from a digital map using ArcGIS [23]. Land use (split into forest, urban, agriculture, grassland, wetland, bareland, and waterside and represented as percentages) within a 1 km-radius circle in the center of the sampling sites was measured from a digital map in ArcGIS [18]. Substrate composition, hydrological variables, and some of the physicochemical variables (e.g., dissolved oxygen (DO), pH, and electrical conductivity) were measured in the field. Water samples were transported in an ice box to the laboratory where measures of biochemical oxygen demand (BOD), total nitrogen (TN), total phosphate (TP), chlorophyll-a (Chl-a), and turbidity were taken according to the APHA method [24]. Substrate composition (in terms of size) was determined according to the methods of Cummins and Lauff [25]. We excluded outlier values during data preprocessing prior to analysis.

### 2.2. Data Analysis

Data analysis was conducted in four steps. First, community indices such as species richness, abundance, Shannon’s diversity index and evenness were calculated. We also analyzed the relationships between taxon abundance and taxon incidence (i.e., the number of sites in which a taxon was observed) using quadratic regression analysis. Species rank-abundance curves were used to represent relative species abundances. Second, we characterized the distribution patterns of the odonate assemblages based on their similarities, using the learning process of a Self-Organizing Map (SOM) [26], which is an unsupervised artificial neural network. SOM consists of input and output layers that are connected by weight vectors. When input data (the odonate assemblages investigated in this study) are fed into the input layer, weighted values are produced. In the output layer, sampling sites are ordinated on the map based on similarities between their species compositions [17,27]. We excluded species that were observed at only one site from the multivariate analysis. The abundances of each species were log transformed using a natural logarithm to reduce variation in species abundances. A value of one was added to the abundance values prior to transformation (i.e., log(abundance + 1)). We used 150 (N = 10 × 15) SOM output units based on the function 5×$\sqrt{\mathrm{number}\;\mathrm{of}\;\mathrm{samples}}$ [28,29]. After the classification of sampling sites using SOM, we further classified the SOM output units using their weight vectors based on a hierarchical cluster analysis using the Ward linkage method and the Bray–Curtis distance measure. SOM is known for its use in the visualization and abstraction of ecological data, and SOM weighted values effectively reflect the assemblage structures of sampling sites in each node [1,30].

We compared the community indices (species richness, abundance, Shannon’s diversity index, and evenness) and environmental variables of clusters (as defined by SOM) using Kruskal–Wallis test. Evenness and the Shannon’s diversity index were calculated for 746 sampling sites (excluding 221 sampling sites that contained only one species). Dunn’s multiple comparisons test was used as a post hoc test if the results of the Kruskal–Wallis test showed significant differences among clusters.

Third, we defined indicator species in each cluster using an indicator species analysis [31]. The indicator value (IndVal) indicates the association between a species and a site cluster [32]. It is defined as the product of the relative abundance and incidence relative observed frequency for each species in each cluster. The IndVal was calculated as follows:

$$A_{ij}=N_{individual_{ij}}/N_{individual_{i}}\;(\mathrm{Relative}\;\mathrm{abundance}\;\mathrm{of}\;\mathrm{each}\;\mathrm{cluster})$$

$$B_{ij}=N_{site_{ij}}/N_{site_{j}}\;(\mathrm{Relative}\;\mathrm{observed}\;\mathrm{frequency}\;\mathrm{of}\;\mathrm{each}\;\mathrm{cluster})$$

$$IndVal_{ij}=A_{ij}\times B_{ij}\times 100\;(\mathrm{Indicator}\;\mathrm{value}\;\mathrm{of}\;\mathrm{each}\;\mathrm{species}\;\mathrm{in}\;\mathrm{each}\;\mathrm{cluster})$$

$$IndVal_{i}=\max\left[IndVal_{ij}\right]\;(\mathrm{Maximum}\;\mathrm{value}\;\mathrm{of}\;\mathrm{indicator}\;\mathrm{value}\;\mathrm{in}\;\mathrm{each}\;\mathrm{species})$$

(i: Species, j: Cluster)

In the actual calculations, the square root of IndVal was used. If the largest $\sqrt{IndVal}$ value for a given species was greater than 5% of the second highest value for that species, the species was designated as an indicator species of the cluster showing the largest $\sqrt{IndVal}$ value [32]. Indicator species analysis used only taxa that were observed in more than five sampling sites (0.5% of all sites).

Finally, we characterized the relationship between odonate assemblages and environmental variables using non-metric multidimensional scaling (NMDS) [33]. We opted to use the weight vectors from the SOM analysis and virtual odonate assemblages rather than raw abundance data, because SOM reduces the dimensions of large datasets, removes noise, and produces smoothing effects [1]. After the ordination of NMDS, community indices and environmental variables were represented in biplots based on the correlation coefficients between their values and the coordinates of the virtual assemblages in the NMDS axes.

To evaluate differences in the habitats of the selected indicator species, we chose two environmental variables in each environmental category based on the coefficient of determination ($r^{2}$) in NMDS. We used indicator species that were observed at more than 200 sampling sites. The preferences of each indicator species for specific environmental variables were evaluated using Kruskal–Wallis test. Dunn’s multiple comparisons test was used as the post hoc test when Kruskal–Wallis test results showed significant differences among indicator species preferences.

All analyses were done in R (https://www.r-project.org/) using packages “stats” [34] for quadratic regression analysis and Kruskal–Wallis test, “PMCMR” [35] for Dunn’s multiple comparisons test, “kohonen” [36] for SOM, “vegan” [37] for cluster analysis and NMDS, and “indicspecies” [32] for indicator species analysis.

## 3. Results

### 3.1. Distribution Patterns of Species

**Figure 2:**
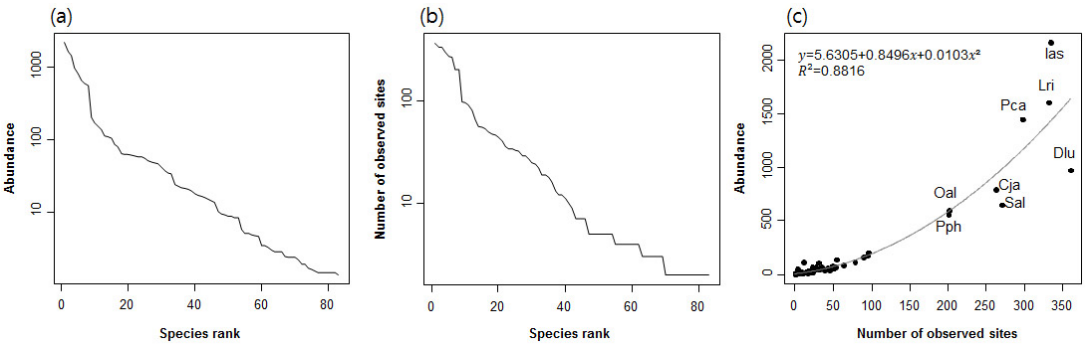
(**a**) Species rank-abundance curve; (**b**) species rank-incidence curve; and (**c**) relationship between species abundance and incidence. Three letters in Figure 2c are abbreviations for the eight dominant species: Ias, *Ischnura asiatica*; Lri, *Lamelligomphus ringens*; Dlu, *Davidius lunatus*; Pca, *Paracercion calamorum*; Sal, *Sieboldius albardae*; Cja, *Calopteryx japonica*; Oal, *Orthetrum albistylum*; Pph, *Platycnemis phillopoda*.

Our dataset consisted of 965 sampling sites, in which a total of 83 odonatan taxa belonging to 10 families were recorded. Nineteen taxa belonged to the suborder Zygoptera and 64 taxa to the suborder Anisoptera. Abundance and incidence both decreased logarithmically as species rank increased (Figure 2a,b). There were abrupt changes in both the abundance and incidence between species ranks 8 and 9, suggesting that there were eight distinctly dominant species. The abundances of species exponentially increased as their incidence increased ($R^{2}$ = 0.882) (Figure 2c). Only eight species, namely *Davidius lunatus*, *Ischnura asiatica*, *Lamelligomphus ringens*, *Paracercion calamorum*, *Sieboldius albardae*, *Calopteryx japonica*, *Orthetrum albistylum*, and *Platycnemis phillopoda*, were observed in numbers greater than 500 individuals at more than 200 sampling sites, while most species were present in numbers less than 250 individuals and were found at fewer than 100 sampling sites. *I. asiatica* was the most abundant species (2162 individuals), followed by *L. ringens* (1603 individuals) and *P. calamorum* (1446 individuals). *D. lunatus* was the most widespread species, having the highest incidence (being recorded at 361 sites), followed by *I. asiatica* (334 sites) and *L. ringens* (332 sites).

### 3.2. Patterns in Odonate Assemblages

**Figure 3:**
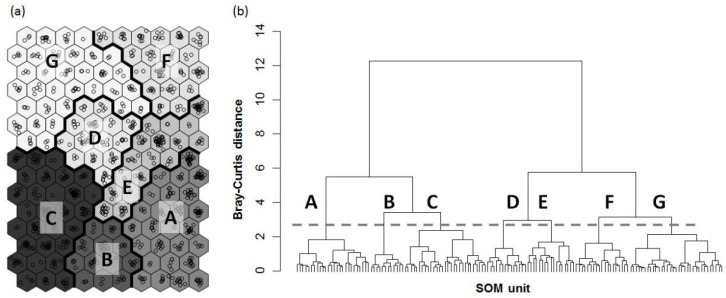
(**a**) Classification of sampling sites using a Self-organizing map (SOM); and (**b**) classification of SOM units based on the dendrogram of hierarchical cluster analysis with Ward linkage methods using Bray–Curtis distances. Weight vectors of SOM units were used in the hierarchical cluster analysis.

The SOM projected 965 sampling sites containing 69 taxa onto the SOM output units, according to the observed similarities in odonate assemblages (Figure 3a). SOM output units were split into seven clusters (A–G) based on the dendrogram generated by hierarchical cluster analysis (Figure 3b). These clusters were further divided into two groups (A–C and D–G). Clusters A–C (located on the lower part of the SOM map) were characterized by species of the suborder Anisoptera, whereas clusters D–G (located in the upper part of the SOM map) were characterized by species of the suborder Zygoptera. *L. ringens*, *S. albardae*, and *D. lunatus* were dominant in clusters A, B, and C, respectively. In cluster D, *P. calamorum* was dominant, and *C. atrata,* which was the ninth-most dominant species at the nationwide scale, was dominant in cluster E. *I. asiatica* was dominant in both clusters F and G.

Indicator species were defined in each cluster based on their IndVal (Table 2). The number of indicator species varied between 1 and 15, among the clusters, and cluster E contained no indicator species whatsoever. Clusters A and B contained two indicators species. *L. ringens* (IndVal statistic = 0.789, *p* < 0.001) in A and *S. albardae* (IndVal statistic = 0.791, *p* < 0.001) in B were indicative species. *D. lunatus* (IndVal statistic = 0.692, *p* < 0.001) characterized cluster C, and *Paracercion hieroglyphicum* (IndVal statistic = 0.259, *p* < 0.001) characterized cluster D with the highest statistical value among the three total indicator species. *Orthetrum albistylum* (IndVal statistic = 0.446, *p* < 0.001) was the most indicative among the four indicator species in cluster F. Eighteen indicator species were present in cluster G, and four species among them were dominant on the nationwide scale. All eight dominant species on the nationwide scale were designated as indicator species.

**Figure 4:**
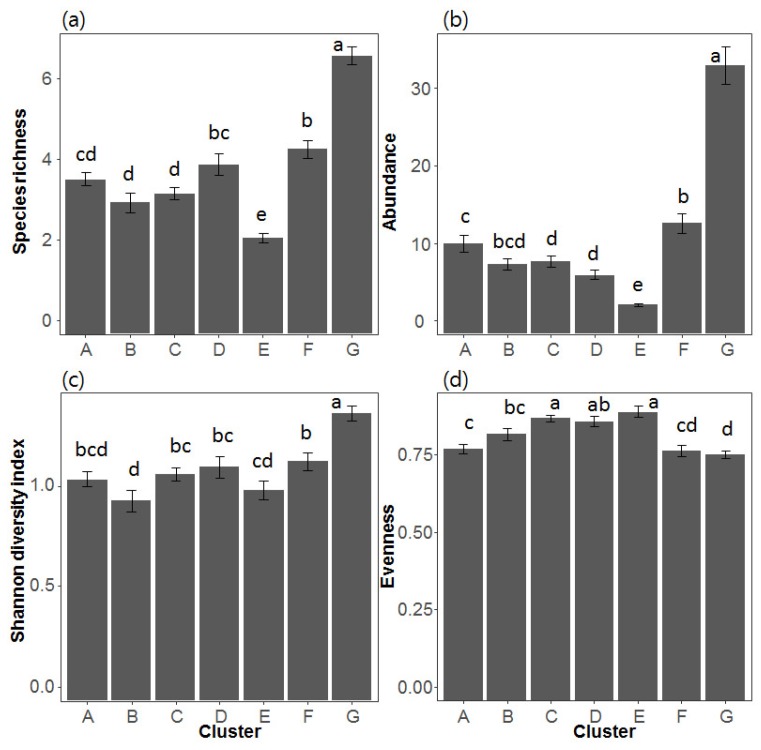
Differences in community indices: (**a**) species richness (number of species); (**b**) abundance (number of individuals); (**c**) Shannon diversity index; and (**d**) evenness. Different letters represent significant differences between clusters based on Dunn’s multiple comparisons test (*p* < 0.05). Error bars indicate standard errors.

**Figure 5:**
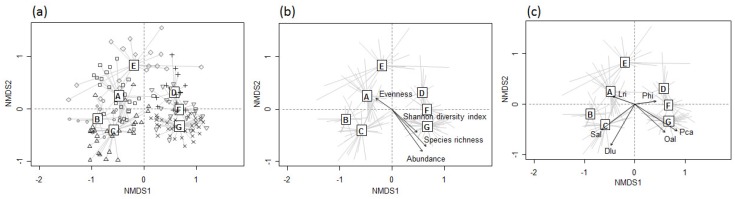
(**a**) The NMDS ordination of odonate assemblages based on the weight vectors of SOM units (stress value: 0.203); (**b**) biplots with community indices; and (**c**) the most significant indicator species in each cluster. Abbreviations for indicator species are given in Table 2.

Community indices were higher in clusters in the upper parts of the SOM map (clusters D–G), and all indices, except evenness, were significantly higher in cluster G, when compared with other clusters (Figure 4). Evenness was significantly lower in cluster G relative to the other clusters. The virtual odonate assemblages split into 150 SOM units were ordinated on the first two axes of the NMDS, reflecting the different clusters produced by the SOM (Figure 5a). SOM units placed in different clusters are indicated by different letters. Community indices and indicator species suitably reflected the differences among clusters (Figure 5b,c).

### 3.3. Differences in Environmental Variables among Different Assemblage Patterns

**Figure 6:**
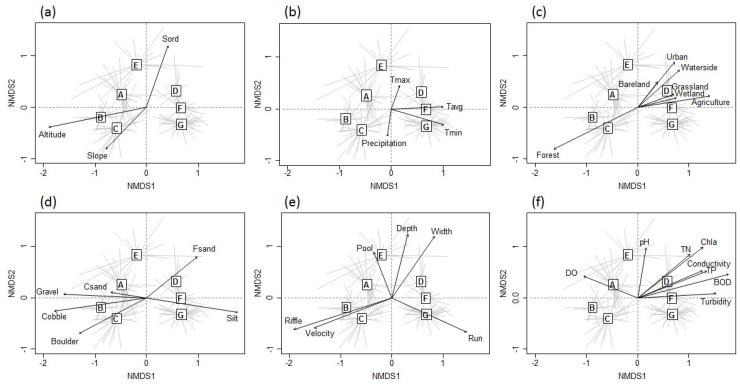
Biplots of environmental variables in six different categories of NMDS ordination: (**a**) Geography; (**b**) meteorology; (**c**) land use; (**d**) substrate composition; (**e**) hydrology; and (**f**) physicochemical water quality. Abbreviations for the variables are given in Table 1.

NMDS ordination characterized differences in the environmental variables that were divided into six categories. Among the geographical variables, altitude was the most correlated with the NMDS axes ($r^{2}$ = 0.577, *p* < 0.01) (Figure 6). Minimum temperature in January was the most contributing variable ($r^{2}$ = 0.536, *p* < 0.01) among all meteorological variables, followed by annual average temperature ($r^{2}$ = 0.485, *p* < 0.01). In the land use category, the proportion of forest was the most determinant ($r^{2}$ = 0.631, *p* < 0.01), followed by the proportion of agricultural land area ($r^{2}$ = 0.3822, *p* < 0.01). Considering substrate composition, the proportion of cobble ($r^{2}$ = 0.565, *p* < 0.01) had the highest relationship with the NMDS ordination axes, followed by that of silt ($r^{2}$ = 0.561, *p* < 0.01). Among the hydrological variables, the proportion of riffle was the most correlated ($r^{2}$ = 0.425, *p* < 0.01), and, among the physicochemical variables, BOD displayed the strongest relationship with the NMDS axes ($r^{2}$ = 0.479, *p* < 0.01), followed by chlorophyll-a ($r^{2}$ = 0.369, *p* < 0.01).

**Figure 7:**
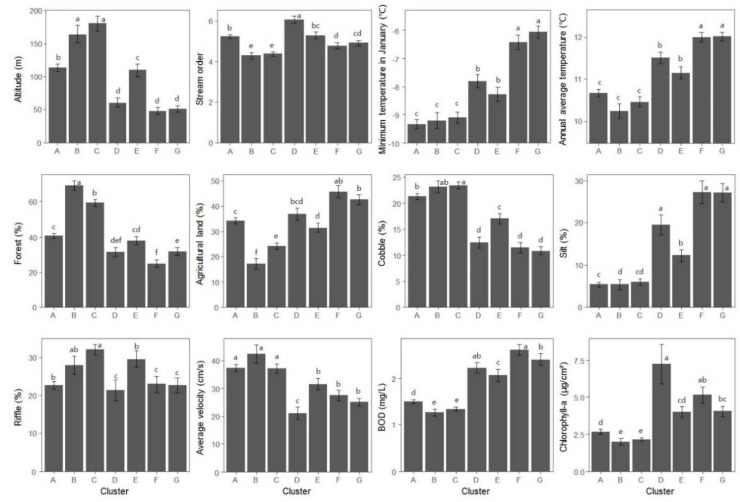
Differences in environmental variables in the seven different clusters. Different letters represent significant differences among clusters based on Dunn’s multiple comparisons tests (*p* < 0.05). Error bar represents the standard error in each cluster.

Environmental factors that were deemed important determinants of odonate assemblage patterns by the NMDS analysis were selected for analysis of their magnitudes across SOM clusters (Figure 7). Among geographical variables, altitude and stream order were selected and were highest in clusters B and C and lowest in clusters D, F and G (Dunn test, *p* < 0.05). Among the meteorological variables, minimum temperature in January and average temperature were selected, and were found to be significantly higher in clusters F and G than in clusters A, B and C (Dunn test, *p* < 0.05). Among the land use variables, forest (%) was the highest in cluster B but the lowest in cluster F (Dunn test, *p* < 0.05). When agriculture (%) was high, forest (%) was frequently low, suggesting some kind of inverse relationship between the two. Among substrate composition, cobble (%) was the highest in cluster C and the lowest in clusters D, F and G. Silt (%) was negatively related to cobble (%). Among the hydrological variables, riffle (%) was the highest in cluster C and current velocity was commonly associated with riffle (%) (Dunn test, *p* < 0.05). Among the physicochemical variables, BOD and chlorophyll-a exhibited similar patterns, being the highest in clusters D–G and the lowest in clusters A–C (Dunn test, *p* < 0.05). Altitude, forest (%), cobble (%), riffle (%), and current velocity were higher in clusters A–C located in the lower parts of SOM map than in clusters D–G, which were located in the upper parts. Conversely, minimum temperature in January, annual average temperature, agriculture (%), silt (%), BOD, and chlorophyll-a were lower in clusters A–C than in clusters D–G. BOD and silt (%) were significantly lower at higher altitudes, and higher when land area was low.

We selected eight dominant indicator species, which showed high abundances and incidences (observed at more than 200 sampling sites) (Figure 2c and Table 2) to evaluate any differences in their environmental conditions (Table 3). Among the eight dominant species, one indicator species in each of the clusters A, B, C, and F were chosen, and four indicator species in cluster G were selected. Species *S. albardae* in cluster B was characterized by high-altitudes, whereas species *O. albistylum* in cluster F, and *P. calamorum*, *Ichnura asiatica*, and *P. phillopoda* in cluster G were more commonly observed in low-altitude areas. *L. ringens* in cluster A and *D. lunatus* in cluster C inhabited habitats with similar environmental conditions to *S. albardae*, but usually at slightly lower altitudes. No dominant indicator species were used in clusters D and E.

## 4. Discussion

Our dataset contains 83 recorded odonatan species from streams and rivers. Among them, eight species, namely *Davidius lunatus*, *Ischnura asiatica*, *Lamelligomphus ringens*, *Paracercion calamorum*, *Sieboldius albardae*, *Calopteryx japonica*, *Orthetrum albistylum*, and *Platycnemis phillopoda*, were dominant with very high incidences (Figure 2). These species were also selected as indicator species. Clusters A–C were dominated by Anisopterans, whereas clusters D–G were characterized by Zygopterans. The differential dominance of these indicator species was mainly due to differences in species habitat preferences as well as differences in their biological and ecological characteristics. For example, Zygopterans typically prefer habitats with a deep and constant water level (e.g., downstream areas) and are weaker fliers over longer distances, whereas Anisopterans primarily inhabit upstream habitats close to forested areas [5].

The distributions of larval odonates is influenced by various scaled environmental factors from microhabitat conditions including water quality and substrate composition to landscape and climate characteristics. For example, NMDS revealed that the altitude, forest ratio (%) in the riparian area, and cobble ratio (%) in substrate composition were the most important environmental factors contributing to differences in odonate assemblage compositions in Korea. Forestation in the riparian area provides important refugia for odonates, allowing them to hide from predators and more effectively hunt prey [38]. The composition of forests and seasonal differences in upstream habitats also affects the structure of benthic macroinvertebrate assemblages including the compositions of odonate assemblages [39,40]. For instance, decreases in canopy cover results in increased sunlight penetration, warmer temperatures, and altered vegetation structure, which can all influence the distribution range, growth rate, and behavior of odonatan species [41]. The presence of vegetation is especially important to some odonatan species such as *C. japonica* [2,42], although most species are not strictly associated with any particular riparian macrophyte [43]. For example, *C. japonica* uses macrophytes as both refugia [16] and in their spawning [44]. *Calopteryx* sp. prefers large substrates when vegetation is sparse, and the influence of substrate size decreases gradually as vegetation density increases [13,45]. *Paracercion hieroglyphicum* characterized cluster D and is found in lentic waters with rich riparian vegetation [44]. Sampling sites in cluster D were mostly downstream sites with open canopies and high chlorophyll-a concentrations, suggesting that high sunlight penetration unites assemblages in cluster D. *Paracercion* requires more sunlight than Anisopteran species such as *O. albistylum* [46,47] as higher temperatures facilitate flight and mating in Zygopteran species. Geographical separation (e.g., habitat fragmentation) is also an important determinant of species distributions [40].

Altitude and the ratio of cobble to silt in substrata were also important in determining odonate assemblage structures in NMDS. Although odonate species are generally known as taxa adapted to warm temperatures and lowland habitats [48], the species *D. lunatus* and *S. albardae* are characteristic of the least disturbed areas and are dominant and representative indicator species in mountainous areas (clusters B and C in Figure 7). Similarly, Harabiš and Dolný [49] reported that altitudinal ranges are associated with dragonfly distributions. Sampling sites in cluster F were mostly from agricultural and urban land areas with low canopy coverage and high turbidity. These environmental characteristics were well characterized by the indicator species *O. albistylum* which is a tactile predator [50] and is tolerant to physicochemical pollution [44]. As they are more competitive than visual predators in turbid environments, they may dominate in these sampling sites. Substrate size is strongly related to habitat condition, influencing organisms’ life cycles and behavior. Many species in Gomphidae are lithophilous and live in association with stony substrata within the lotic habitats. Meanwhile, species such as *L. ringens*, an indicator species in cluster A, preferred finer substrate sizes as they burrow into the substrate to hide from predators [16]. Our study also showed that cluster G was characterized by silty substrata, as indicated by the identification of the silt-preferring *Calopteryx* sp. which needs vegetation as an indicator species [9,45].

## 5. Conclusions

Our research investigated the distribution patterns of odonate species in streams and factors influential in the determination of odonate assemblage compositions on a nationwide scale in South Korea. A total of 83 odonatan taxa belonging to 10 families were recorded in our dataset consisted of 965 sampling sites. Among them, eight species, *Davidius lunatus*, *Ischnura asiatica*, *Lamelligomphus ringens*, *Paracercion calamorum*, *Sieboldius albardae*, *Calopteryx japonica*, *Orthetrum albistylum*, and *Platycnemis phillopoda*, displayed distinctly high abundance and incidence. The abundances of species exponentially increased as their incidence increased. Sampling sites were classified into seven different clusters based on differences in odonate assemblage compositions and environmental conditions based on SOM and cluster analysis. Sampling sites were clearly differentiated between being dominated by members of the suborder Anisoptera or the suborder Zygoptera. Among the eight dominant species, *S. albardae* in cluster B was characterized by high-altitudes, whereas *O. albistylum* in cluster F, and *P. calamorum*, *Ichnura asiatica*, and *P. phillopoda* in cluster G were more commonly observed in low-altitude areas. *L. ringens* in cluster A and *D. lunatus* in cluster C inhabited habitats with similar environmental conditions to *S. albardae*, but usually at slightly lower altitudes. Indicator species in each cluster had different habitat preferences. Particularly, differences of altitude, forest (%), and cobble (%) in substrata were mainly responsible for variation in assemblage compositions and the determination of indicator species. Finally, our results showed the importance of habitat heterogeneity by demonstrating its effect on odonate assemblage patterns.

## Figures and Tables

**Table 1:** Environmental characteristics of study areas.

Category	Variable (Unit)	Abbreviation	Mean	SE *	Range
Geography	Stream order	Sord	5.0	0.05	1.0–9.0
	Altitude (m)		109.8	3.67	0.0–680.4
	Slope (°)		1.3	0.14	0.0–38.8
Meteorology	Total annual precipitation (mm)	Precipitation	1242.4	3.64	955.7–1773.9
	Annual average temperature (°C)	Tavg	11.1	0.05	5.7–15.4
	Maximum temperature in July (°C)	Tmax	27.5	0.04	23.1–29.1
	Minimum temperature in January (°C)	Tmin	–8.2	0.09	–14.3–3.1
Land use	Urban (%)		12.2	0.56	0.0–93.4
	Forest (%)		40.8	0.91	0.0–100.0
	Agricultural land (%)	Agriculture	32.2	0.71	0.0–94.9
	Grassland (%)		4.1	0.15	0.0–36.2
	Wetland (%)		2.7	0.10	0.0–28.1
	Bareland (%)		3.3	0.15	0.0–50.5
	Waterside (%)		4.8	0.23	0.0–99.5
Substrate composition **	Silt (%)		11.3	0.60	0.0–100.0
	Fine sand (%)	Fsand	23.1	0.55	0.0–91.9
	Coarse sand (%)	Csand	18.9	0.26	0.0–65.0
	Gravel (%)		21.2	0.32	0.0–65.0
	Cobble (%)		17.8	0.35	0.0–50.0
	Boulder (%)		7.6	0.31	0.0–60.0
Hydrology	Water width (m)	Width	57.0	3.03	1.0–1400.0
	Water depth (cm)	Depth	30.7	0.45	9.9–149.2
	Current velocity (cm/s)	Velocity	32.0	0.73	0.0–106.6
	Percentage of riffle (%)	Riffle	19.5	0.62	0.0–100.0
	Percentage of run (%)	Run	58.8	0.98	0.0–100.0
	Percentage of pool (%)	Pool	21.8	0.93	0.0–100.0
Physiochemistry	Dissolve oxygen (mg/L)	DO	8.8	0.04	3.2–12.9
(Water quality)	Biochemical oxygen demand (mg/L)	BOD	1.9	0.04	0.6–10.8
	Total Nitrogen (mg/L)	TN	2.6	0.04	0.7–10.8
	Total Phosphate (mg/L)	TP	0.1	0.00	0.0–1.1
	Chlorophyll-a (μg/m^2^)	Chl-a	3.6	0.17	0.6–90.1
	pH		7.8	0.01	6.3–9.2
	Electric conductivity (μS/cm)	Conductivity	224.6	6.74	22.0–2626.0
	Turbidity (NTU)		12.0	0.45	0.0–94.3

* SE: Standard error; ** Silt: <0.0625 mm; Fine sand: 0.0625–2 mm; Coarse sand: 2–16 mm; Gravel: 16–64 mm; Cobble: 64–256 mm; Boulder: >256 mm.

**Table 2:** Indicator species of each cluster based on the IndVal.

Suborder	Family	Species	Frequency	Cluster	Stat *	*p*-Value
Anisoptera	Gomphidae	*Lamelligomphus ringens*	332	A	0.789	<0.001
Anisoptera	Gomphidae	*Ophiogomphus obscura*	64	A	0.332	<0.001
Anisoptera	Gomphidae	*Sieboldius albardae*	271	B	0.791	<0.001
Anisoptera	Corduliidae	*Macromia amphigena*	54	B	0.246	0.003
Anisoptera	Gomphidae	*Davidius lunatus*	361	C	0.692	<0.001
Zygoptera	Coenagrionidae	*Paracercion hieroglyphicum*	33	D	0.259	<0.001
Anisoptera	Corduliidae	*Macromia manchuria*	43	D	0.219	0.003
Zygoptera	Lestidae	*Lestes sponsa*	6	D	0.135	0.044
Anisoptera	Libellulidae	*Orthetrum albistylum*	202	F	0.446	<0.001
Anisoptera	Libellulidae	*Pantala flavescens*	23	F	0.224	0.003
Anisoptera	Libellulidae	*Sympetrum parvulum*	6	F	0.198	<0.001
Anisoptera	Libellulidae	*Sympetrum kunckeli*	11	F	0.139	0.046
Zygoptera	Coenagrionidae	*Paracercion calamorum*	298	G	0.684	<0.001
Zygoptera	Coenagrionidae	*Ischnura asiatica*	334	G	0.661	<0.001
Zygoptera	Calopterygidae	*Calopteryx japonica*	263	G	0.548	<0.001
Zygoptera	Platycnemididae	*Platycnemis phillopoda*	201	G	0.452	<0.001
Anisoptera	Aeshnidae	*Anax parthenope*	79	G	0.431	<0.001
Zygoptera	Platycnemididae	*Copera annulata*	95	G	0.387	<0.001
Anisoptera	Libellulidae	*Deielia phaon*	49	G	0.344	<0.001
Anisoptera	Libellulidae	*Crocothemis servilia*	90	G	0.32	<0.001
Anisoptera	Libellulidae	*Libellula quadrimaculata*	28	G	0.261	<0.001
Zygoptera	Coenagrionidae	*Enallagma cyathigerum*	33	G	0.241	0.002
Zygoptera	Calopterygidae	*Atrocalopteryx atrata*	96	G	0.219	0.029
Anisoptera	Aeshnidae	*Anax nigrofasciatus*	15	G	0.210	0.003
Anisoptera	Gomphidae	*Shaogomphus postocularis*	32	G	0.200	0.009
Anisoptera	Libellulidae	*Orthetrum lineostigma*	39	G	0.180	0.024
Anisoptera	Corduliidae	*Epitheca marginata*	21	G	0.165	0.021

* Stat: statistical value of IndVal in square root.

**Table 3:** Differences (mean ± SE) of environmental variables among selected indicator species in each cluster. Different letters present significant differences between species based on Dunn’s multiple comparison test (*p* < 0.05). We selected eight dominant indicator species which showed occurrence frequency which were observed at more than 200 sampling sites, as shown Figure 2c.

Cluster	Species	Environmental Variables					
		Altitude (m)	Temperature (°C) *	Forest (%)	Cobble (%)	Riffle (%)	BOD (mg/L)
A	*Lamelligomphus ringens*	110.0 (4.3) ^b^	−8.9 (0.1) ^c^	44.2 (1.2) ^b^	21.0 (0.5) ^b^	20.6 (0.8) ^a^	1.5 (0.0) ^c^
B	*Sieboldius albardae*	147.2 (6.4) ^a^	−9.3 (0.2) ^c^	58.8 (1.5) ^a^	23.4 (0.5) ^a^	24.6 (1.2) ^a^	1.2 (0.0) ^e^
C	*Davidius lunatus*	150.8 (7.4) ^b^	−9.0 (0.1) ^c^	55.4 (1.4) ^a^	21.7 (0.5) ^b^	25.2 (1.0) ^a^	1.4 (0.0) ^d^
F	*Orthetrum albistylum*	50.9 (3.2) ^d^	−6.9 (0.2) ^a^	31.0 (1.7) ^d,e^	11.7 (0.7) ^d^	11.6 (1.1) ^c^	2.4 (0.1) ^a^
G	*Paracercion calamorum*	61.9 (3.9) ^d^	−6.9 (0.1) ^a^	32.7 (1.4) ^d^	12.7 (0.6) ^d^	11.4 (0.9) ^c^	2.2 (0.1) ^b^
G	*Ischnura asiatica*	54.0 (3.0) ^d^	−6.7 (0.1) ^a^	28.0 (1.3) ^e^	12.0 (0.5) ^d^	12.9 (1.0) ^c^	2.4 (0.1) ^a^
G	*Calopteryx japonica*	90.9 (4.8) ^c^	−7.7 (0.2) ^b^	38.9 (1.5) ^c^	17.3 (0.6) ^c^	18.3 (1.0) ^b^	1.6 (0.1) ^c^
G	*Platycnemis phillopoda*	62.2 (4.6) ^d^	−7.2 (0.1) ^a^	33.1 (1.7) ^d,e^	12.2 (0.7) ^d^	11.4 (1.1) ^c^	2.3 (0.1) ^a,b^

* Minimum temperature in January.
